# Supplementary material for: Are carbon emissions trading and green financial instruments synergistic? -Comprehensive quantitative research based on content analysis
Source: PLoS One. 2024 Mar 7;19(3):e0298601. doi: 10.1371/journal.pone.0298601 (PMC10919601; doi:10.1371/journal.pone.0298601)
Supplement: S1 Data — (ZIP) [file pone.0298601.s001.zip › Data/地级市Z_F工作报告.zip!/║╙─╧/╨φ▓2╩╨/└┤╫╘╣1⁄2╓┌║┼í╛┬φ┐╦╩2╛▌═°í┐_2013.docx]

各位代表：

现在，我代表市人民政府，向大会作工作报告，请予审议，并请各位政协委员提出意见。

一、2012年工作回顾

2012年是本届政府任期的第一年。在省委、省政府和市委的坚强领导下，市政府团结带领全市人民，深入贯彻科学发展观，认真落实党的十八大精神，坚持加快发展、科学发展不动摇；坚持破解瓶颈、克难攻坚不懈怠；坚持用好载体、抓好抓手不折腾；坚持创新机制、推动落实不空谈，较好地完成了市六届人大一次会议确定的各项目标任务。全市生产总值1739亿元，增长12.3%，增速居全省第1位；社会消费品零售总额482.4亿元，增长16.3%，增速居全省第2位；规模以上工业增加值956.6亿元，增长17.4%，增速居全省第4位；固定资产投资1111.5亿元，增长23.9%；地方公共财政预算收入90.4亿元，增长21.8%；城镇居民人均可支配收入19685元，增长9.3%；农民人均纯收入9819元，增长11.2%。2012年我们主要做了以下工作：

（一）全力以赴稳定经济增长。完善政策措施，加强经济运行调节，经济下行趋势得到有效遏制。以项目建设为抓手，实施“1241”投资促进计划，120个重点项目完成投资477亿元，带动全社会固定资产投资1155.7亿元；深刻领会、准确把握宏观调控政策，争取上级项目资金12.4亿元，为历年来最多。加强企业服务，定期召开重点企业政策咨询会，帮助企业解难题，问题办结率达100%；认真落实各项税费减免政策，为企业减、免、退税32.4亿元；深入开展政银企对接，达成贷款意向305.6亿元，新增贷款153.6亿元，利用资本市场融资90.5亿元。4家企业在天交所成功挂牌，主营业务收入超百亿元的企业达到6家，规模以上工业利润居全省第2位。

（二）全面加快新型城镇化建设。中心市区124个城建重点项目完成投资58亿元，51个城中村改造项目完成投资74.8亿元，建设安置房252.8万平方米。七一路文峰路（隧道）工程、西湖公园改造等一批重大项目竣工投用。许昌新区主、副中心建设加快推进。许昌至新郑机场快速通道如期开工，许禹快速通道建设顺利，新元大道东延建成通车，城市组团联系更加紧密。开工建设中心镇27个、新型农村社区68个，其中36个社区已基本建成，“三化”协调科学发展试验区建设全面启动。深入推进文明城市创建，荣获国家节水型城市称号，城市管理水平持续提升，卫生城市届满复审顺利通过。

（三）积极推动产业结构调整。深入实施产业集聚区“2623”提升计划、服务业提升计划，加大自主创新体系建设力度，加强节能减排和环境保护，经济增长速度、质量和效益同步提升。10个产业集聚区规模以上工业主营业务收入1700亿元，8个省级产业集聚区个均固定资产投资居全省第1位。三大主导、四大特色、五大战略性新兴产业主营业务收入分别突破1800亿元、800亿元和260亿元。服务业增加值增长9.3%，专利申请量和授权量居全省第3位，新增国家高新技术企业8家，许继集团特高压交流输电项目荣获国家科技进步奖特等奖，中原电气谷核心区成为省级高新技术产业开发区，黄河集团获得省长质量奖。万元生产总值能耗、主要污染物减排目标顺利完成。

（四）扎实做好“三农”工作。加快建设高标准粮田“百千万”工程，粮食总产283万吨，实现“九连增”。积极推进十大农业产业化集群培育工程，襄城生猪产业集群完成投资6亿元，鄢陵花木产业集群建设稳步提升。大力发展现代农业，流转土地达142.4万亩，规模经营面积500亩以上的农业公司达178家。不断完善农村基础设施，新解决农村居民安全饮水25.5万人，新增节水灌溉面积11万亩，发展和改善有效灌溉面积12万亩，完成农田水利“最后一公里”工程建设36万亩、林业生态建设21.6万亩。

（五）不断深化改革开放。列入台账的30家企事业单位已完成改革改制18家，城市公交公司化改造基本完成；医疗卫生体制改革取得阶段性成果，省定3年医改任务圆满完成。着力破解发展瓶颈，建立完善了规划两级审批、土地资源保障联席会、拆迁工作流程、政银企洽谈会等卓有成效的工作机制。成功举办第七届豫商大会、第六届三国文化周等招商活动，引进省外资金260亿元，实际利用外资4.4亿美元、增长22.5%，实现出口16亿美元、增长20.2%。扎实推进开放体系建设，与国家开发银行、国家电网等12家省直厅局和央企签署了战略合作协议或备忘录，许昌出入境检验检疫局成功获批，许昌海关申报工作取得实质性进展。

（六）持续改善人民生活。加大民生投入，“十项民生工程”全面完成。全市财政用于民生的支出达127.1亿元，占公共财政预算支出的71.2%。全面落实促进就业创业各项政策，城镇新增就业9.1万人，新增农村劳动力转移就业9.7万人，成为全省唯一获得“全国创业先进城市”称号的省辖市。提高社会保障水平，城乡居民社会养老保险制度实现全覆盖，企业退休人员养老金、城乡低保金、五保供养标准持续提高。新开工建设保障性安居工程14406套，建成13500套。编制完成并启动了清潩河流域水环境综合整治行动计划。优先发展教育事业，完成公办幼儿园建设项目40个、中小学校舍安全工程项目138个，市区小学重点项目、中职基础能力建设项目有序推进，河南农大许昌校区实现秋季招生，许昌电气职业学院一期工程建成投用。文化、卫生、体育、人口、广播电视等各项社会事业全面发展。

（七）积极推进民主法制。自觉接受人大、政协和社会监督，办理人大代表议案建议和政协委员提案318件。行政审批事项精简至192项，办理时限压缩到6.5天。加强审计监督，进一步完善了重点项目资金监管等工作机制。完善惩治和预防腐败体系，严肃查处了一批违法违纪案件。扎实推进平安许昌创建工作，深入开展信访问题排查和信访积案化解活动，持续加强安全生产隐患治理，保持了全市大局和谐稳定。国防动员、人防、民兵预备役建设和对台工作进一步加强，民族宗教、外事侨务、地方史志、档案、统计、地震、气象等工作都取得了新的成绩。

这些成绩的取得，是省委、省政府和市委正确领导的结果，是市人大、市政协监督支持的结果，是全市广大干部群众共同奋斗的结果。在此，我代表市人民政府，向全市人民，向人大代表、政协委员，向驻许部队、武警官兵、公安民警表示崇高敬意！向所有关心、支持许昌发展的各界人士表示衷心感谢！

在总结成绩的同时，我们也清醒地看到，全市经济社会发展还存在一些突出矛盾和问题：影响经济发展的不确定、不稳定因素依然较多，城镇化水平不高，需求动力不足，经济企稳回升基础不稳；要素约束加剧，经济结构性矛盾依然突出，服务业发展水平还不够高，调结构转方式步伐仍需加快；公共服务体系还不够健全，城乡居民收入整体水平不高，社会管理创新面临新压力；政府职能和作风转变还不到位，一些干部忧患意识不强、创新精神不足、能力素质不高、工作作风不优。对此，我们将高度重视，采取有力措施认真加以解决。

二、2013年目标任务

今年是全面贯彻落实党的十八大精神和实施《中原经济区规划》的开局之年，是实施“十二五”规划承前启后的关键一年，是为全面建成小康社会奠定坚实基础的重要一年。总体来看，我市经济社会发展具备较多有利条件和积极因素，但也面临不少风险和挑战，我们必须抢抓机遇、开拓进取、克难攻坚、科学发展，努力取得经济社会发展新成效。

今年政府工作的总体要求是：以党的十八大精神为指导，全面贯彻落实中央和省委、省政府决策部署，围绕市委提出的目标要求，持续促发展，切实转方式，着力惠民生，争创新优势，促进“三化”协调、“四化”同步，保持经济平稳较快发展、社会和谐稳定，努力在实现“中原梦”的伟大征程中不断开创许昌率先崛起、富民兴许新局面。

今年我市经济社会发展的主要预期目标是：生产总值增长10%；地方公共财政预算收入增长12%；工业增加值增长13%；全社会固定资产投资增长22%；社会消费品零售总额增长16%；外贸进出口总额增长15%；实际利用外商直接投资增长12%；城镇居民人均可支配收入、农民人均纯收入实际增长10%；城镇化率提高2个百分点；居民消费价格涨幅3.5%左右；人口自然增长率控制在6.4‰以内；新增城镇就业6.5万人；节能减排完成省定目标。

实现上述目标，做好今年政府工作，需要坚持以下几个原则：一是更加注重稳定增长。牢牢把握稳中求进的工作总基调，积极采取扩大需求的政策措施，发挥好消费、投资对经济增长的基础作用和关键作用，稳定扩大出口规模，确保实现今年经济社会发展目标任务。二是更加注重发展质量。以转变经济发展方式为主线，以提高质量效益为中心，促进“三化”协调、“四化”同步，实现尊重经济规律、有质量、有效益、可持续的发展。三是更加注重改善民生。强化民本意识，着力稳定和扩大就业，加大民生投入，加强城乡社会保障体系建设，促进公共服务均等化，不断提高人民生活水平。四是更加注重改革创新。坚持用改革的办法破除体制机制障碍，用创新的举措破解发展难题，着力培育形成发展的新活力、新动力、新体系、新优势。五是更加注重优化环境。坚持优化“软”环境与改善“硬”环境并重，加快基础设施、产业载体、开放平台等硬件建设，改进完善政府服务体系和流程，提升公共服务水平，营造更加优良的发展环境，着力实现“一优带百通”。

三、2013年重点工作

按照市委经济工作会议部署，围绕总体目标，今年突出抓好八项重点工作。

（一）着力强化项目支撑，持续促进经济平稳增长。充分发挥项目建设对扩需求、调结构、转方式的载体作用，提高运作能力和水平，推进项目建设实现新突破。保持投资适度较快增长。大力实施“15512”投资促进计划，突出抓好150个重点项目，完成年度投资超过500亿元，拉动全市固定资产投资超过1200亿元。这些项目涵盖了工业、现代服务业、社会事业、交通电力、农林水利、城镇建设等六大领域，既是保增长的关键，也是调结构的抓手，应集中力量、强力实施。切实改进项目推进措施。加强项目分类指导和监管，对保障性住房、市科技馆等公共投入的重点项目，逐一研究，抓好投资模式、合同条款、招投标、财政评审、审计、备案等关键环节；对许昌电梯产业园、中原国际农产品物流产业基地一期工程等社会投资项目，集中研究，加大协调力度，为项目建设创造有利条件；对其他社会投资项目，大力支持，积极引导，加快建设。努力提高项目储备质量。以国家批准《中原经济区规划》和建设郑州航空港经济综合实验区为契机，以提升服务业比重、信息化水平为重点，科学谋划一批符合国家产业政策、切合本地发展实际、提振公众信心的重大优质项目。对重大前期项目，充分论证、主动对接，力争纳入省、国家计划。

（二）着力新型城镇化引领，持续推进城乡发展一体化。充分发挥新型城镇化“一发动全身”的综合带动效应，加快城镇化进程，提升城镇化质量。完善提升中心市区。完成总体规划修编，有序推进重点区块开发建设和基础设施完善提升，实施好总投资354.9亿元的134个城建重点项目和49个城中村棚户区改造项目，分别完成年度投资65.9亿元、57.2亿元。优化城区功能布局，积极稳妥开发建设城市综合体。加快商务中心区和特色商业区建设，促进人口与产业有机集聚；加大城区路网、背街小巷、市政设施等建设改造力度，完善城市功能。加快许昌零距离换乘中心、许昌至新郑机场快速通道、长途汽车站和公交停保场建设，放大交通区位优势。巩固提升国家文明城市创建成果，抓好国家园林城市复检、全国无障碍建设城市、全国综治优秀市等创建工作。实施好数字城管二期扩建、城市街景景观综合整治、城区夜景亮化等工程，提升城市信息化、精细化、科学化管理水平。加快建设许昌新区。实施好主导区总投资260亿元的60个重点项目，完成年度投资70亿元。科学编制商务中心区、贸易加工区、中原电气谷拓展区等各项规划，加快完善规划体系。围绕主、副中心建设，着力抓好芙蓉湖、芙蓉广场、北海游园等基础设施项目。加快商务中心区发展，扎实推进企业总部建设，确保移动公司营业用房、芙蓉国际城市综合体等20个项目全部开工建设，谋划实施会展中心、演艺中心等特色服务业项目。统筹推进社区建设和社会事业发展，抓好学校、医院、公租房等设施建设，完善服务功能。加快发展组团城市。全面完成许禹快速通道建设，启动省道103线禹州至襄城段改建等6条国、省道建设，加快永昌大道、中原路等道路建设，完善“一中心五组团”快速交通体系。推动5个组团城市加快老城区连片改造和新城区整体建设，加快城镇基础设施扩容提升，增强人口承载能力。同时，积极推进30个中心镇建设，着力完善基础设施和公共服务设施，因地制宜发展特色产业，引导农民向镇区转移，推动中心镇成为县域经济社会发展的关键支撑。扎实建设新型农村社区。落实“两个优先、五化十有”，加快社区基础设施和社会公益事业的配套完善。突出农民主体，尊重农民意愿，保障农民权益，加快安置步伐，调动群众参与建设的积极性，促进新型农村社区积极稳妥健康发展。

（三）着力新型工业化主导，持续提升经济发展水平。深入实施创新驱动发展战略，坚持承接产业转移与培育自身内生动力并举，加快推进产业结构调整和集聚集群发展，切实提高发展质量和效益。加快产业集聚区发展。深入实施产业集聚区提升工程，重点抓好200个亿元以上产业转型升级项目，带动产业集聚区完成固定资产投资750亿元，实现规模以上工业企业主营业务收入突破2000亿元。依托中原电气谷核心区，做好规划，争创国家高新技术开发区。加快产业集聚区功能性、生产性和生活性基础设施建设，合理布局、集中规划建设公租房性质的职工公寓，不断提升产业集聚区的承载力、竞争力。推动产业优化升级。实施100个省级工业结构调整项目和30个研发与产业化项目，力争三大主导、四大特色、五大战略性新兴产业主营业务收入分别突破1900亿元、900亿元、300亿元。实施十大产业链发展计划，推进延链补链，增强产业链条整体竞争力，不断提升产业集群规模和水平。实施服务业提升计划，持续推进“3615”现代物流业提速工程，大力发展钧瓷、生态等文化产业集群，完成招商银行入驻开业，改造提升餐饮住宿、商贸物流、交通运输等传统服务业，扶持发展信息服务、创意设计、社区养老等新型业态。促进企业做大做强。加快企业上市步伐，力争恒源发制品成功上市，5家企业在天交所挂牌。深入实施企业创新能力培育科技工程，抓好重大科技专项和自主研发平台建设，消除规模以上工业企业“零专利”，力争新增5家以上高新技术企业。大力实施“质量立市、品牌兴业”、商标和标准化战略，力争1家企业获得省长质量奖，新增中国驰名商标1件。深入实施大企业（集团）、小巨人企业、高新技术企业培育计划，完善重点企业服务制度，力争主营业务收入超百亿元企业达到7家。深入推进“两化融合”。抓好智慧城市、无线城市建设，加大信息化平台、信息传输系统、通信服务提升工程等基础设施建设，促进信息网络技术广泛应用，鼓励引导有条件的企业全面提高数字化、网络化、自动化和智能化水平。

（四）着力打牢农业基础，持续促进“三化”协调发展。认真落实强农惠农富农政策，加快转变农业发展方式，大力推动农业公司化经营和农业产业化集群发展，不断提升农业现代化水平。稳定粮食生产。抓好高标准粮田“百千万”建设工程，提高粮食综合生产能力，力争粮食总产稳中有增。推进“五县十乡”万亩高产示范片和“千亿斤”粮食生产项目，全年新建高标准粮田39万亩。加强科技、信息、农机、气象、流通等服务体系建设，促进农业生产各环节、全过程专业化发展。坚持以家庭承包经营为基础，支持新型农民合作组织、农业社会化服务组织多形式和多层次发展，逐步构建新型农业经营体系，保障农民合法权益，持续增加农民收入。推动集群发展。以国家农业科技园区建设为依托，大力发展农业产业化集群。提升许昌鄢陵花木产业集群，抓好河南建业集团鄢陵绿色基地建设，发展高档绿化苗木和鲜花切花，全年新增花木面积5万亩。推动禹州、长葛、襄城生猪产业集群加快形成规模，抓好“千场百区标准化提升工程”和畜牧园区建设，畜牧业产值突破150亿元。扩大烟草、蔬菜、中药材、蜂产品等特色产业优势，全年蔬菜复种面积100万亩，烟叶和中药材种植面积分别稳定在15万亩和40万亩。突出抓好河南襄城烟草产业集群里川示范区规划建设，推动烟草、生猪产业集群联动发展。有序推进土地流转，流转面积达到148万亩，新增农业合作社200家。完善基础设施。加大防洪工程建设力度，扎实推进6条中小河流、33座小型水库和10座中型水闸除险加固工程及重点岁修工程。突出抓好灌区节水改造、防洪除涝、河道治理等11个水利项目建设，提高水资源保障和防灾减灾能力。加强农田水利建设，完成农田水利“最后一公里”工程建设39万亩。加强农村水电路气等设施建设，完成农村电网改造升级投资2.06亿元，新解决农村居民安全饮水20万人，改造农村公路400公里，新建农村户用沼气5000口。加强扶贫开发和23个村整村推进工作，新解决2.72万人脱贫问题。加快南水北调配套工程建设，做好移民后期帮扶工作。

（五）着力深化改革创新，持续增强发展活力动力。把改革创新作为加快转变经济发展方式的关键举措，积极构建有利于科学发展的体制机制，释放发展的巨大潜力、源头活力和持久动力。推进企事业单位改革改制。扎实进行20家企事业单位改革改制，认真解决历史遗留问题，加快劣势资本退出，推动企业战略重组、产权转让、依法破产、转企改制和内部改革。按照内部监管、行业自律原则，巩固改制成果，促进企业发展。加快重点领域改革。加快电广传媒、文博旅游等文化团体整合重组，推动文化产业做大做强。抓好新农合支付制度改革工作，完善基本药物制度和基层医疗机构运行新机制，统筹实施县级公立医院综合改革，促进基本公共卫生服务均等化。推进林权制度改革，提高集体林地家庭承包率和发证到户率。深化资源型产品价格改革，以价格杠杆推动结构调整。创新要素保障机制。加强政银企合作，整合优质资产资源，做大投融资平台。强化多元融资，开展中小企业区域集优票据融资，推动城投债二期、集合债、企业债发行，实现全年社会融资总量260亿元，力争新增贷款180亿元，直接融资80亿元。加强征信体系建设，严厉查处非法集资行为，切实防范和化解金融风险。科学推进征地拆迁，紧紧依靠群众，确保公开、公正、公平。严格落实建设用地政策，科学制定土地收储、出让、利用等计划，加强土地综合整治，积极推进人地挂钩试点工作，探索节余建设用地指标流转，促进土地集约节约利用。大力开展定向、定岗和订单式培训，强化人力资源支撑。坚持煤电运联调联动，加强电力需求侧管理，提高生产保障能力。

（六）着力扩大对外开放，持续增创发展新优势。把开放作为应对复杂局面、促进改革发展“一举求多效”的综合性举措，靠扩大开放带动全局、推动转型、跨越发展。加大招商引资力度。实施“6116”招商引资行动计划，围绕承接产业转移，谋划推介600个重点招商项目，合同引进市外资金1100亿元，突出抓好60个重点招商项目。创新招商方式，围绕十大产业链发展计划，依托10个产业集聚区，瞄准世界500强、央企、行业龙头企业，办好第13届中国·中原花木交易博览会、第7届三国文化旅游周、第8届禹州钧瓷文化节等重大招商活动，突出招大引优，着力开展专业化、集群式招商。完善招商引资奖励机制、招商项目快速落地机制，进一步调动重点企业、相关部门、各级领导班子、沿海地区商协会的积极性，切实提高项目履约率、开工率和资金到位率。扩大开放领域。积极引导外资更多投向主导特色产业、战略性新兴产业、现代农业、基础产业、城乡建设等领域，集中推介一批金融保险、商贸物流、文化旅游项目，力争服务业开放招商实现新突破。积极推介许昌新区、产业集聚区基础设施、公共服务设施项目，吸引外资参与城镇化和新型农村社区建设。引进优质教育、医疗资源，吸引外资来我市设立医疗机构、投资文化产业。借助郑州航空港经济综合实验区，认真谋划建设许昌承接区，承接临空产业转移。优化开放环境。建立服务企业和项目落地开工的快速通道，加快完善招商引资信息处理和受理服务中心、联审联批中心、投诉权益保护中心，构建“三位一体”招商引资全程服务平台。建立全市外商投诉联席会议制度，开展投资环境评价评议活动，强化投资环境的督查问责，创造高效的政务环境、市场环境。完善交通物流体系和各类产业载体配套设施，加快大通关建设，力争许昌出入境检验检疫局上半年挂牌运行，许昌海关申建工作取得新突破。

（七）着力加强生态文明建设，持续打造美丽许昌。把生态建设放在更加突出的位置，推进绿色、循环、低碳、可持续发展，为人民谋求长远福祉。扎实推进节能减排。抓好重点行业、重点区域和列入省千家节能低碳行动企业的节能减排工作，加快实施节能改造、节能惠民产品等重点节能工程。倡导“低碳生活”，加强公共领域节能与新能源汽车示范推广试点工作，申报新能源示范城市。大力发展循环经济，加快建设大周镇国家“城市矿产”示范基地。加强生态系统建设。注重生态文明建设与城市建设有机融合，突出抓好城区、高速公路及廊道绿化、西部山区生态体系建设和矿区生态修复工程，巩固提高林业生态建设水平，完成造林任务19.9万亩。加快禹州市第三污水处理厂等12个污水处理项目建设，扎实推进清潩河流域水环境综合整治工程，深入开展农村环境连片综合治理，做好环境空气质量新标准监测的准备工作。强化生态文明制度保障。坚持规划环评制度，实行最严格的耕地保护、水资源管理、环境保护制度，建立生态补偿制度。坚持“五禁止、五限制”，加强项目节能准入管理，加快淘汰落后产能。严格实行主要污染物排放总量预算管理，开展节能量、碳排放权、排污权有偿使用交易。加强环境监管，健全生态环境保护责任追究制度。

（八）着力提升民生福祉，持续促进社会和谐稳定。更加重视保障和改善民生、发展社会事业，扎实推进“十项民生工程”，让人民共享改革发展成果。积极扩大就业。健全城乡公共就业服务体系，加大就业创业扶持力度，重点做好城镇困难人员、农村转移劳动力、高校毕业生、退役军人就业工作，新增城镇就业6.5万人、农村劳动力转移就业6.5万人以上。加快推进全民技能提升工程，开展各类培训20万人次以上。强化社会保障。加大社会保险扩面征缴力度，完善养老保险转移接续办法，提高社会保险经办能力和服务水平，加快县级综合福利中心和社会养老机构基础设施建设。继续提高企业离退休人员养老金、城乡低保、五保供养标准和优抚对象待遇水平。稳定低生育水平，全面提高出生人口素质。认真落实计生家庭奖扶政策，完善社会救助制度，重视解决弱势群体困难。加强保障性住房建设管理，新建保障性住房1万套，逐步满足中低收入群体基本住房需求。办好社会事业。促进义务教育均衡发展，推进义务教育学校标准化和教育信息化建设，全面启动43个新型农村社区学校项目，完成6所市区小学改扩建、35所公办幼儿园建设、农村中小学和义务教育薄弱学校校舍改造任务。大力发展职业教育，积极推进许昌电气职业学院二期、许昌卫生学校扩建等项目，不断完善现代职业教育体系。切实改善群众就医条件，加快实施东城区三甲医院等5个重点项目和73个重大疾病防控、县级医院和乡镇卫生院改扩建项目，扩大新农合重大疾病保障范围，认真做好重大传染病防控救治工作。大力实施文化旅游惠民工程，抓好11个文化旅游产业重点项目建设，扎实做好许昌塔文化博物馆、市科技馆等文化事业项目，引导各类文化活动走进基层。广泛开展全民健身运动，办好许昌市第七届运动会。维护社会稳定。创新社会管理，深入开展平安许昌创建，加强社会管理综合治理，做好流动人口和特殊人群的服务管理，提高应急处突能力，严厉打击刑事犯罪。坚持“打非治违”常态化，加强对煤矿、交通、食品药品等重点行业领域的专项检查，确保安全生产形势持续稳定。实行“发放明白卡、签订承诺书、建立黑名单”制度，加快形成全链条、无缝隙的安全生产监管体系。严格落实信访稳定风险评估、领导接访和包案制度，积极探索管用的信访处置机制，深入开展矛盾纠纷排查化解，切实解决信访群众实际困难。加强社会公德、职业道德、家庭美德、个人品德教育，打牢和谐稳定基础。

继续加强国防和民兵预备役工作，促进军民融合式发展。重视和支持工会、共青团、妇联、工商联等人民团体工作。切实做好外事、侨务、港澳、对台工作。全力做好第三次经济普查工作，继续做好民族、宗教、地方史志、新闻出版、人事、审计、统计、档案、地震、气象、人防、社科研究等各项工作。

四、切实加强政府自身建设

把改进工作作风、提升能力水平作为加强政府自身建设的关键，认真贯彻中央“八项规定”和省委、市委落实意见，深入推进“两转两提”，着力建设人民满意的政府。

（一）推进依法行政，建设法治政府。落实依法治国基本方略，推进依法行政，用法治思维和方式深化改革、推动发展、化解矛盾、维护稳定。认真办理人大代表议案、建议和政协委员提案，自觉接受人大法律监督、政协民主监督、舆论监督和社会监督。完善依法行政各项制度，规范行政执法，不断提高政府工作制度化、规范化和程序化水平。

（二）强化宗旨意识，建设服务政府。强化执政为民理念，进一步完善为企业、为群众、为基层服务的各项制度，提高服务质量和效率。继续精简行政审批事项，切实转变政府职能，加强政务服务体系建设，加快政府职能向创造良好发展环境、提供优质公共服务、维护社会公平正义转变。深入推进政务公开，为人民群众提供优质便捷高效服务。

（三）转变工作作风，建设效能政府。坚持一切从实际出发，在部署工作中出实招，在落实工作中求实效，不尚空谈，不做虚功，不图虚名。精简会议文件，改进会风文风，减少检查评比，力戒形式主义、官僚主义，集中精力办成一批大事、办好一批实事、办妥一批难事。坚持务实高效，雷厉风行，推动各项工作提速增效。切实提高执行能力，优化工作流程，完善工作机制，做到政令畅通、令行禁止。

（四）坚持从严治政，建设廉洁政府。认真落实党风廉政建设责任制，建立健全惩治和预防腐败体系，加强监察、审计监督，规范权力运行，防止权力寻租。厉行勤俭节约，反对铺张浪费，严控行政经费等一般性支出，把有限的钱花在建机制、增效益上，用在保障和改善民生上。严厉查处违纪违法案件，严肃惩治不正之风和腐败行为，以实际行动赢得群众的信任与支持。

各位代表！时代赋予重任，人民寄予厚望，我们深感责任重大、使命光荣。让我们在省委、省政府和市委的正确领导下，紧紧依靠全市人民，凝心聚力，锐意进取，求真务实，克难攻坚，努力在中原经济区建设中率先崛起、富民兴许，为全面建成小康社会、奋力实现“中原梦”做出新的更大贡献！
